# Chromosomal Breakage in Fanconi Anemia and Consanguineous Marriages: A Social Dilemma for Developing Countries

**DOI:** 10.7759/cureus.10440

**Published:** 2020-09-14

**Authors:** Fakeha Siddiqui, Saqib Ansari, Akbar Agha, Nadeem Nusrat, Saima Munzir, Saira Shan, Anny Hanifa, Tasneem Farzana, Mehwesh Taj, Munira Borhany, Zeeshan Hussain, Muhammad Nadeem, Tahir Shamsi

**Affiliations:** 1 Internal Medicine, Dow University of Health Sciences, Karachi, PAK; 2 Hematology, National Institute of Blood Diseases and Bone Marrow Transplantation, Karachi, PAK; 3 Hematology, Dow University of Health Sciences, Karachi, PAK

**Keywords:** aplastic anemia, fanconi anemia, consanguinity

## Abstract

Introduction

A clear picture of the prevalence of Fanconi anemia is not known due to limited studies and research of the subject. This study will detect the frequency of positive chromosomal breakage in pediatric aplastic patients and provide the evidence-based guidelines which help in consideration of appropriate treatment and awareness to the society.

Methods

A total of 104 aplastic anemia patients were recruited of age <18 years whose samples were tested for chromosomal breakage with mitomycin C (MMC). History of consanguinity between parents were documented for all the patients referred to us.

Result

Out of 104 diagnosed aplastic anemia patients, 35 (33.7%) patients were found to be Fanconi positive. Mean age of all hypoplastic patients for aplastic anemia and Fanconi anemia was 10.7 ± 4.5 and 10.6 ± 3.5, respectively. Male preponderance was found to be higher (64, 61.5%) as compared to females (40, 38.5%) in aplastic patients. The male to female ratio was observed as 2.5:1 in Fanconi patients while 1.3:1 in non-Fanconi aplastic patients. Parental consanguinity was observed in 33 (94.2%) with Fanconi anemia.

Conclusion

Fanconi anemia accounts for significant number of patients with hypoplastic bone marrow, therefore consanguineous marriages should be avoided through mass education in Pakistan.

## Introduction

Marriages between blood relatives seem to be one of the major causes of genetic diseases. Many undiagnosed cases of mental retardation and congenital abnormalities may have some underlying genetic abnormality mostly autosomal recessive mutation. Fanconi anemia is one of those autosomal recessive disorder linked in rare cases, with chromosomal instability [1,2]. It is the fate of many carrier families which become conspicuous after consanguineous marriages.

Pakistan is one of those countries where consanguineous marriages are favored culturally. This is one of the major reasons for the high prevalence of inherited genetic disorders. Fanconi anemia is an autosomal recessive disorder frequently associated with bone marrow failure, congenital anomalies and malignancies. It is the most frequent cause of inherited aplastic anemia [3].

A clear picture of the prevalence of Fanconi anemia is not known due to limited studies and research on the subject. One of the initial reports of a study conducted in Pakistan in 2008, shows that the prevalence of Fanconi anemia was found to be 16.6% in patients suffering with aplastic anemia [4]. While a study conducted in India in 2014 showed the prevalence rate of 13.1%. In aplastic patients, affected cells characteristically show hypersensitivity to DNA cross linking agents like mitomycin C, cisplatinum, deoxybutane leading to high frequency of chromosomal breakage. This genomic instability leads to the development of a diagnostic test [5].

Patients with Fanconi anemia usually present between 5 to 10 years of age with diverse symptoms and manifestation. This includes mental retardation, congenital anomalies like small ears, microphthalmia, short stature, absence of thumb and other systemic visceral and somatic abnormalities [6]. Altered skin pigmentation and/or café au lait spots, abnormal male gonads, microcephaly, structural renal defects, low birth weight, developmental delay are also observed in many patients [7]. However, 25 to 40% of Fanconi anemia patients have a normal phenotype [8].

The estimated incidence of Fanconi anemia is three cases per million newborn per year and it is more slightly commonly seen in males than females. The male to female ratio is 1.3/1. The carrier frequency however is 1/300 in the USA and western Europe. While in other parts of the world it is about 1/90-1/100; this includes Ashkenazi Jews, Spanish Gypsies and South African Afrikaners [9].

It is important to rule out genetic disorders for accurate diagnosis and appropriate management. Consanguineous marriages are practiced culturally in the developing world and serve as a favorable medium for the prevalence of various genomic instability syndrome [10-12].

In Pakistan, the diagnosis of many inherited disorders with low prevalence rate is underestimated. They are either underdiagnosed due to lack of knowledge and financial constraint or misdiagnosed due to overlapping of presenting symptoms [1].

It is to be noted that thalassemia is not just one hematologic condition that is transferred from affected parent of a consanguineous couple. Various hematologic and non-hematologic conditions are genetically influenced. These diseases are in consensus with burden on society and a challenge to be encountered in the twenty first century. Therefore, it is necessary to be focused and give proper attention to these overlooked problems in order to prevent the families from further cousin marriages and subsequent generations.

Although during the last decade, lot of work has been done by different organizations and social media to educate and increase public awareness. This has mostly been associated with thalassemia, however many other undiagnosed disorders are prevalent in the developing countries where the actual prevalence rate of the disease is not known due to the lack of knowledge and limited research on the subject [1,7].

Fanconi anemia is one of those underestimated problems that is prevailing in the developing countries like Pakistan and India, and is a social burden for these countries [13]. According to a study the clinical manifestation is highly variable and overlapping of symptoms with those observed in other bone marrow failure syndromes which make its diagnosis very difficult [14,15]. Hence we believe that by knowing the frequency of Fanconi anemia associated with cousin marriages, it will help the policy makers to plan the strategies to control the disease by counseling the families to stop further consanguineous marriages (with the carrier family) and prevent the future generation from acquiring this dangerous genetically acquired medical disease.

## Materials and methods

This cross-sectional study was performed in the National Institute of Blood Disease, Karachi, Pakistan during the period from August 2014 to September 2015. We recruited 104 aplastic patients under 18 years of age. A medical appointment with a complete history and examination was performed. An informed consent was taken for each patient and data was recorded. History of consanguinity was recorded for each patient as well. Patients included were with bone marrow failure (hypoplastic/aplastic bone marrow aspirates/biopsy). For all cases, marrow hypoplasia/aplasia was defined based on standard Camitta’s criteria [3,5].

Chromosomal breakage analysis by MMC method

**Figure 1:**
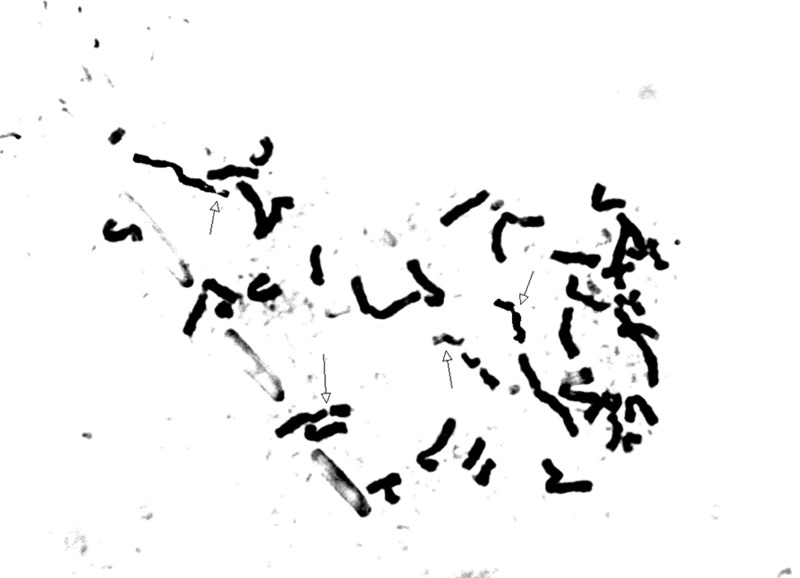
Mitomycin C-induced chromosomal aberrations in our patient with Fanconi anemia showing chromosomal breaks, acentric fragments, and chromatid break

About 3-4 ml of peripheral blood samples were collected in heparinized container from each patient. Chromosomal spreads were obtained from phytohemagglutination stimulated 72 hours culture of peripheral blood lymphocytes using standard protocols. Cultures were set up in duplicates for mitomycin C (MMC) stress test by adding 40 ng/mg MMC at the time of initiation of culture [4]. Replicate sets of untreated cultures were kept as controls. Metaphase stained with Giemsa was analyzed from each culture. Slides were prepared and codified for the analysis of chromosomal aberrations [3,4,13]. Each cell was scored for chromosome number and for the numbers and types of structural abnormalities (Figure 1).

**Figure 2:**
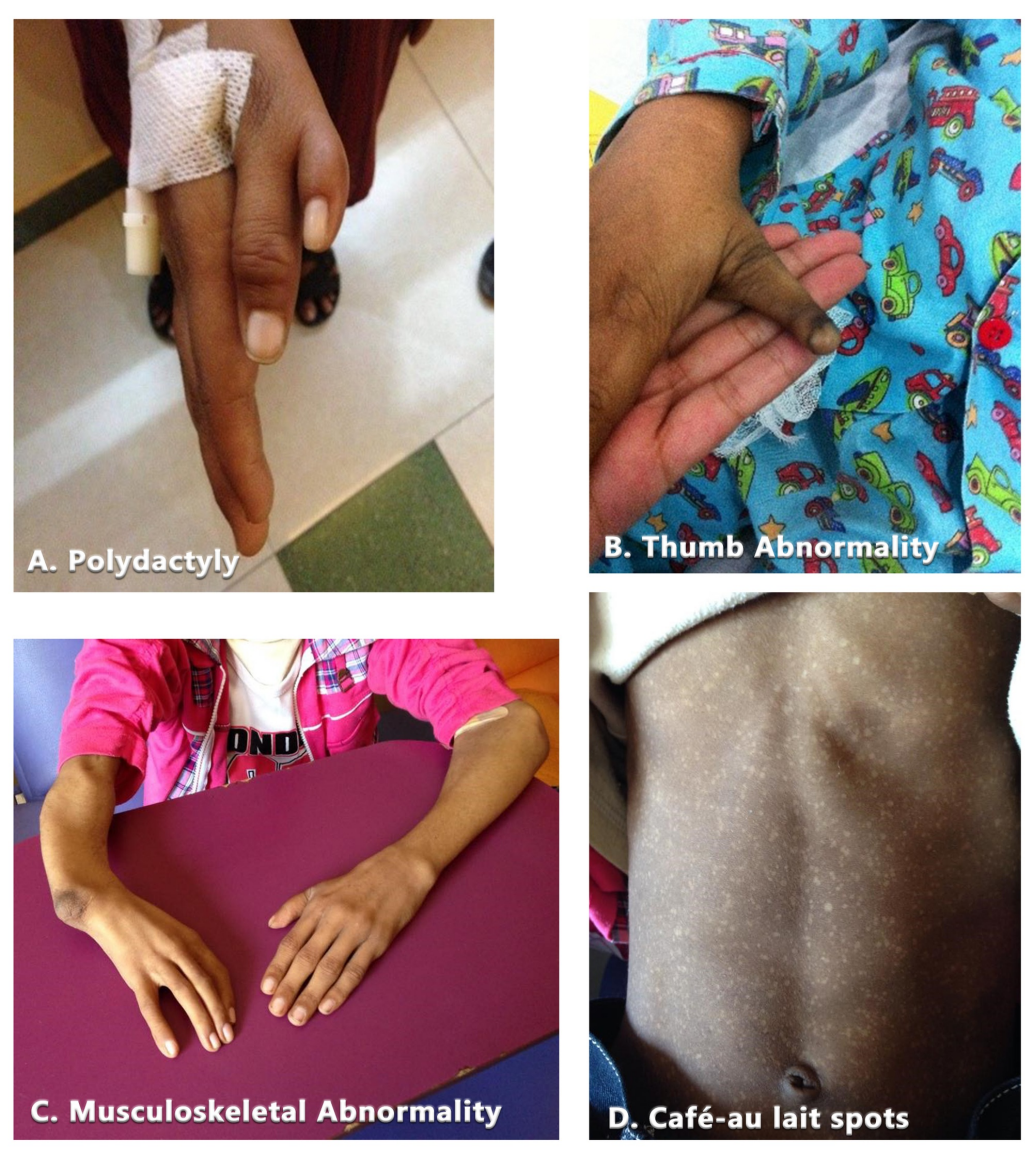
Physical abnormalities in children included in our study

The children included in our study showed a wide range of physical abnormalities typical of Fanconi anemia, as shown below (Figure 2A-2D).

## Results

The patients belonged to different cities of Pakistan. The maximum aplastic patients (34%) belonged to the interior Sindh, a province in Pakistan. The age of patients was below 18 years. Out of 104 diagnosed aplastic anemia patients, positive chromosomal breakage test indicating Fanconi anemia positive was observed in 35 (33.7%) aplastic patients, whereas 69 (66.3%) were found to be Fanconi anemia negative (Table 1).

**Table 1:** Age distribution of aplastic patients (n = 104)

	Age groups	Aplastic patients		Total
		Fanconi -ve Anemia (n = 69) n (%)	Fanconi +ve Anemia (n = 35) n (%)	
1	1-10	38 (76%)	12 (24%)	50
2	>10	31 (57%)	23 (43%)	54

Mean age of all hypoplastic patients was 10.7 ± 4.5 and 10.6 ± 3.5 for total aplastic patients and Fanconi anemia, respectively. Male preponderance was found to be higher (64, 61.5%) as compared to females (40, 38.5%) in aplastic patients. The male to female ratio was observed as 2.5:1 in Fanconi patients while 1.3:1 in non-Fanconi aplastic patients. Severe aplastic anemia was found to have a higher frequency (63, 60.6%) as compared to very severe aplastic anemia (26, 25%) and mild aplastic anemia (15, 14.4%) (Table 2).

**Table 2:** Distribution of patients on the basis of severity of aplastic anemia

Diagnosis	n (%)
Non-severe Aplastic Anemia	15 (14.4%)
Severe Aplastic Anemia	63 (60.6%)
Very Severe Aplastic Anemia	26 (25%)

Parental consanguinity among Fanconi positive was observed in 33 (94.28%) out of total 35 patients while parental consanguinity among Fanconi negative patients was recorded as 40 (57.9%) out of total 69 patients (Table 3).

**Table 3:** Frequency of parental consanguinity in aplastic patients

Consanguineous couple	n (%)	p-value
Fanconi positive (35)	33 (94.28%)	0.00013
Fanconi negative (69)	40 (57.9%)	

## Discussion

Fanconi anemia is a rare genetic disorder affecting all ethnic groups found approximately one in 360,000 births in Asia [14,16]. Genes responsible for this disease could be either autosomal recessive or X linked. The primary mechanism of all mutated genes is that they do not allow DNA repair mechanisms to work correctly [14]. This mutation leads the patients to a high propensity to develop hematological as well as other solid tumors [12]. The disease is diagnosed by chromosomal breakage analysis method based on the technique described by Auerbach in 1981, where DNA damage, breakage and rearrangements is promoted by using DNA cross linking agents like deoxybutane or mitomycin C [4,5].

The incidence of Fanconi anemia is unknown due to very few number of research studies and a lack of data availability. Moreover, due to limited data, the results are not epidemiologically verified. The present study was conducted with 104 pediatric patients presented to the hematology department of National Institute of Blood Disease.

The results of our study showed that the frequency of positive chromosomal breaks in total aplastic patients was recorded as 33.7%. This result is in contradiction with a study conducted in Karachi in 2008, where the incidence was found to be 16.6% [1]. This result is also in contrast with the study results conducted by Chowdhry et al. in India, where they reported the frequency as 13.1% [6]. For all of the rest of aplastic anemia types, the phenotypic and laboratory findings are integral including skeletal, nail abnormalities, cardiac anomalies and sequence occurrence of cytopenias for amegakaryocytic thrombocytopenia, pure red cell aplasia, Diamond-Blackfan anemia and dyskeratosis congenita. The patients in our study were ruled out phenotypically for these disorders.

Among the individuals of this study, the frequency of aplastic anemia was found to be higher between the age group of 11-18 years, which is analogous to a study result showing the peak incidence of aplastic anemia between age group 11-20 years [1]. The mean age group of aplastic anemia and Fanconi anemia was found to be 10.7 ± 4.5 and 10.6 ± 3.5, respectively. The result of our study is consistent with the literature reported from Thailand, China and Korea, where majority of patients fall under the same age group [13,17-19].

The frequency of aplastic anemia (34%) and Fanconi anemia (37.1%) was found to be higher in Sindhi cast as compared to other ethnicities. This could have been possible as maximum number of patients during our study period presented from Karachi and interior Sind. The earlier literature reported in west showed that the geographical variation is considered mainly due to environmental factors [19,20]. However, this is a small group, large group study may confirm the findings.

In this study, male preponderance was found to be higher in aplastic anemia (61.5%) as well as in Fanconi anemia (71.4%). The ratio of male to female patients for aplastic anemia and Fanconi anemia was analyzed as 1.6:1 and 2.5:1, respectively. This observed ratio is in variance with the ratio reported from west [21,22], but inconsistent with the data reported from the Asian countries particularly Pakistan [1,6,23].

In our study, majority of patients fall under the category of severe aplastic anemia (60.6%), very severe aplastic anemia (25%) and non-severe aplastic anemia (14.4%). Most of the Fanconi anemia patients were found to have severe aplastic anemia (51.4%). These results are similar to the data reported elsewhere [1,6]. However, Goswami et al. reported the frequency of severe and non-severe aplastic anemia as 33.33% and 57.14%, respectively, which is in contradiction with our study results [24].

A study conducted in Jordan reported that the high rate of parental consanguinity seems to cause 32% increase in the proportion of autosomal recessive disorders [15]. Our study showed the frequency of parental consanguinity between aplastic patients to be 73%. The parental consanguinity among Fanconi anemia patients was found to be as high as 94.2% while among non-Fanconi anemia, it was recorded as 57.9%. These significant results are in contradiction with the results reported earlier in the literature where a study reported earlier in India showed the consanguinity to be 36.4% in aplastic population [25]. Altay et al. analyzed the association of consanguinity within 65 Turkish aplastic patients. They reported that the frequency found out to be 46% in non-Fanconi aplastic patients while 78% in Fanconi anemia patients [26].

A report from Israel had given a frequency of 40% consanguinity among aplastic patients [27]. In France only two cases were diagnosed with a positive parental consanguinity out of 147 aplastic patients (1.3%) [28].

Our results emphasize the importance of high incidence of Fanconi anemia among the consanguineous couples as well as early diagnosis of Fanconi anemia, both due to its complex nature of the disease as well as its clinical exhibition being a part of other diseases [29]. The clinical features overlap in one syndrome or other making its diagnosis very difficult [17]. In our study, induced chromosomal breakage analysis could diagnose 35 Fanconi anemia in 104 aplastic patients. Although the severity of disease was reported high amongst these patients and the anemia was already well established leading to bone marrow failure, but screening Fanconi anemia amongst siblings of affected person and early diagnosis can help identify silent cases [6,18].

Transmission of genetic mutations for diseases is one of the greatest fears amongst parents. Prevention of inheritance of such devastating disorder is a great challenge [30]. Delayed diagnosis of Fanconi anemia is a serious threat to the patients and their families. High risk couples should be given appropriate genetic counseling as the recurrence risk for Fanconi anemia is 25% [22,23]. An early diagnosis with appropriate management can help the affected families to find compatible donor for bone marrow transplant [25]. It is necessary to spread the awareness about these overlooked problems in order to prevent the families from social burden and mental trauma.

## Conclusions

A delay in diagnosing Fanconi anemia can cause serious damage to the patients and their families. A prompt diagnosis and appropriate management can help them to find suitable compatible donor for bone marrow transplant. High risk families (families with an affected child having consanguinity positive) should be identified early and provided genetic counselling. Couples should be given prenatal diagnosis as the recurrence risk for this autosomal recessive condition is 25%. It is necessary to be focused and give proper attention to these overlooked problems in order to prevent the families from social burden and mental trauma. The strong association of Fanconi anemia with consanguinity is an alarming situation as this disease has been under diagnosed. The reason for this includes: Lack of expertise in Fanconi anemia testing, poor health infrastructure, low socioeconomic status and lack of affordability for the expensive test, high prevalence of consanguineous marriages and lack of awareness and exposure regarding this rare disease which makes it more difficult to estimate the actual prevalence of the disease.

Although we cannot change the culture and system of our society but such studies will help to council and educate the families where the member of the family has already been diagnosed in order to reduce the morbidity, mortality and financial constraint in Pakistani society.
